# Identifying Psychosocial and Ecological Determinants of Enthusiasm In Youth: Integrative Cross-Sectional Analysis Using Machine Learning

**DOI:** 10.2196/48705

**Published:** 2024-09-12

**Authors:** Roberta M Dolling-Boreham, Akshay Mohan, Mohamed Abdelhack, Tara Elton-Marshall, Hayley A Hamilton, Angela Boak, Daniel Felsky

**Affiliations:** 1 Temerty Faculty of Medicine University of Toronto Toronto, ON Canada; 2 Krembil Centre for Neuroinformatics Centre for Addiction and Mental Health Toronto, ON Canada; 3 Center for Industrial Relations and Human Resources University of Toronto Toronto, ON Canada; 4 School of Epidemiology and Public Health Faculty of Medicine University of Ottawa Ottawa, ON Canada; 5 Institute for Mental Health Policy Research Centre for Addiction and Mental Health Toronto, ON Canada; 6 Dalla Lana School of Public Health University of Toronto Toronto, ON Canada; 7 Campbell Family Mental Health Research Institute Centre for Addiction and Mental Health Toronto, ON Canada; 8 Department of Psychiatry University of Toronto Toronto, ON Canada; 9 Institute of Medical Science University of Toronto Toronto, ON Canada

**Keywords:** subjective well-being, Ontario Student Drug Use and Health Survey (OSDUHS), machine learning, Shapley additive explanations (SHAP), extreme gradient boosting (XGBoost), psychosocial, ecological, determinants, enthusiasm, mental health, well-being, youth, public health, student, self-reported

## Abstract

**Background:**

Understanding the factors contributing to mental well-being in youth is a public health priority. Self-reported enthusiasm for the future may be a useful indicator of well-being and has been shown to forecast social and educational success. Typically, cross-domain measures of ecological and health-related factors with relevance to public policy and programming are analyzed either in isolation or in targeted models assessing bivariate interactions. Here, we capitalize on a large provincial data set and machine learning to identify the sociodemographic, experiential, behavioral, and other health-related factors most strongly associated with levels of subjective enthusiasm for the future in a large sample of elementary and secondary school students.

**Objective:**

The aim of this study was to identify the sociodemographic, experiential, behavioral, and other health-related factors associated with enthusiasm for the future in elementary and secondary school students using machine learning.

**Methods:**

We analyzed data from 13,661 participants in the 2019 Ontario Student Drug Use and Health Survey (OSDUHS) (grades 7-12) with complete data for our primary outcome: self-reported levels of enthusiasm for the future. We used 50 variables as model predictors, including demographics, perception of school experience (i.e., school connectedness and academic performance), physical activity and quantity of sleep, substance use, and physical and mental health indicators. Models were built using a nonlinear decision tree–based machine learning algorithm called extreme gradient boosting to classify students as indicating either high or low levels of enthusiasm. Shapley additive explanations (SHAP) values were used to interpret the generated models, providing a ranking of feature importance and revealing any nonlinear or interactive effects of the input variables.

**Results:**

The top 3 contributors to higher self-rated enthusiasm for the future were higher self-rated physical health (SHAP value=0.62), feeling that one is able to discuss problems or feelings with their parents (SHAP value=0.49), and school belonging (SHAP value=0.32). Additionally, subjective social status at school was a top feature and showed nonlinear effects, with benefits to predicted enthusiasm present in the mid-to-high range of values.

**Conclusions:**

Using machine learning, we identified key factors related to self-reported enthusiasm for the future in a large sample of young students: perceived physical health, subjective school social status and connectedness, and quality of relationship with parents. A focus on perceptions of physical health and school connectedness should be considered central to improving the well-being of youth at the population level.

## Introduction

Mental illnesses among youth account for up to 70% of all disability-adjusted life years [1] in high-income countries, and 20% of Canadian youth experience symptoms of mental illness, with many needing medical intervention [2]. Although there is no universally agreed upon definition of youth, the traditional definition refers to those aged 10-19 years [3] but can vary across cultures, often reflecting social responsibilities, and encompass those up to the age of 25 years [4]. Over 75% of mental disorders occur before the age of 25 years [5], with this period being crucial for developing the skills and habits necessary for lifelong mental well-being. Mental illness in youth can lead to long-lasting negative physical, psychological, and social impacts on individuals and their families [6,7]. Therefore, much work has been dedicated to understanding the behavioral, experiential, environmental, and demographic correlates of mental illness in youth. However, mental health is not defined as merely the absence of illness [8], and much less effort has been spent on identifying the multifactorial correlates of wellness [9].

Understanding adolescent well-being at a population level rather than evaluating mental illness in clinical settings is critical for implementing universal mental health promotion measures. Population-wide studies on well-being thus far highlight the influence of social connectedness [10,11] and competence in social, occupational, and academic domains [11]. Other studies suggest that depression and anxiety symptoms [12,13] and chronic disease [12] have important influence on well-being.

Enthusiasm for the future (herein “enthusiasm”) is an established component of mental wellness [14] and is among the attitudes gauged with the Positive and Negative Affect Schedule scale [15] to measure positive affect. Enthusiasm is strongly correlated with mental wellness [16,17] and is able to independently predict other aspects of well-being, including life satisfaction, positivity, personal growth, improved social connections, and enhanced self-purpose [18]. However, the multifactorial and complex nature of youth well-being [1] makes the determination of policy and programming with the most potential for impact extremely challenging. Thus, understanding the major components of youth well-being, and therefore, potential targets of public health intervention call for methodologies that inherently manage such complexities.

Machine learning is an excellent tool for analyzing complex, multifactorial data across several domains, including public health, diagnostics, treatment, and prognosis [19]. Interpretable methods such as extreme gradient boosting (XGBoost) [20] and Shapley additive explanations (SHAP) [21] gaining popularity, are nonlinear interactive models capable of identifying important features. This approach has been successfully applied to population-based studies to better understand multifactorial contributors to mental illness [22]. However, few studies have applied machine learning to population-based data to investigate mental well-being [23]. In this study, we used a gradient-boosted tree-based machine learning algorithm to better understand multifactorial contributors to self-reported enthusiasm in 13,661 participants from the 2019 Ontario Student Drug Use and Health Survey (OSDUHS). We then applied SHAP analysis to explain the resulting models.

## Methods

### Survey Data Collection

Data from the 2019 cycle of OSDUHS were used for analyses. OSDUHS is a biennial cross-sectional voluntary survey conducted among students in grades 7-12 attending publicly funded schools within Ontario, with the goal of collecting data on their physical, mental, and social well-being and the prevalence of self-reported risk behaviors such as gambling and drug use. OSDUHS data have been widely used to help direct policy decision-making, public programming aimed at supporting youth, and setting priorities for enhancing the health of youth [24]. Administered since 1977, OSDUHS uses a 2-stage cluster sample design based on a random selection of schools stratified by region and school level (elementary and secondary) and a random selection of classes within each school [24].

The 2019 cycle of OSDUHS, administered in 263 schools across 47 school boards within Ontario, had a sample of 14,142 students. The cycle administered 4 versions of the survey: 2 versions for elementary students (grades 7-8) and 2 for secondary school students (grades 9-12). Each version consisted of a set of core questions that were common across all 4 surveys regarding demographics, perception of school experience, family life, substance use, physical activity, hours of sleep, and other physical and mental health indicators. Responses to these core questions constituted the set of features used in our analysis. Survey weights were not used in our analysis. Further details about the 2019 OSDUHS are publicly available [24].

### Definition of the Primary Outcome

Our primary outcome of interest was student self-reported level of agreement to the question “I feel enthusiastic about my future”[25], answered on a 4-point Likert scale (0: strongly disagree to 3: strongly agree). This single Likert-type item of self-reported enthusiasm was used as an outcome given its simplicity and interpretability when building a machine learning model, while also being cognizant of its limitations in being able to capture the multidimensional nature of the well-being construct. This question was posed to all students, maximizing the included sample, and enthusiasm is a known contributor to well-being and positive affect [14,15]. Furthermore, the Likert scale is a fundamental psychometric tool used to quantify qualitative attributes of the human experience [26]. A Likert scale style survey is easier to administer and minimizes the participant burden, increasing the likelihood of survey completion. Of the total sample, 13,661 student participants provided a response to self-reported enthusiasm and were included in our modelling (481/14,142, 3.5% missing). Raw values and 2 transformations of this variable were used to fit 3 different models, as described in the Statistical Modelling section below. Likert-style outcomes have been used in previous machine learning classification studies [27,28].

### Processing of Model Inputs

A complete set of 50 variables was selected from the aforementioned common core questions to maximize the number of student responses that could be included for model training and testing. A range of data types were used in the analysis, including numeric, ordinal, and unordered categorical. Variables such as household composition (family members present in the home environment), racial background, and geographic region within Ontario were one-hot encoded (the process of splitting a categorical variable into multiple binary dummy variables with either yes or no membership), a method often used for categorical variables in tree-based learning models. Ordinal variables were treated as numeric (the machine learning algorithm selected for analysis was XGBoost, later described, which is a nonlinear model and therefore makes no scalar assumptions about levels of ordinal variables). Some variables such as the 10-item responses to “How often did you drink alcohol in the last 12 months?” were collapsed into binary responses (yes vs no) corresponding to whether any alcohol was consumed in the last 12 months. These collapsed variables were created by the OSDUHS team and were provided to us in the original OSDUHS data set [24]. This was the case for all questions pertaining to substance use and was done based on the very low response rates—largely in categories of substance use—for extreme values of some variables.

Additional processing involved re-encoding and collapsing of categories for ease of interpretation. First, any response option that could be interpreted as a “no” was collapsed into a common category. For example, the 2 response options “use internet, but not social media,” and “don't use the internet” were combined to “don’t use social media” for the question “How many hours do you typically spend on social media?” Second, the weight of the student was considered to be the average of the 2-kg range options provided to students. Third, the many categories for language spoken were condensed to categories of “English only,” “French only,” “English and French only,” and “other multiple languages.” All encoded ordered categorical variables were shifted to start with a value of zero. Finally, the remaining categorical variables were one-hot encoded (0=no, 1=yes for category membership), with “don’t know” or “unsure” responses being treated as missing. Crucially, XGBoost was designed to manage missing values by learning appropriate tree branches during the training process and implementing a default mechanism for evaluating new data, which negates the need for a priori imputation. A complete descriptive list of the input variables used in our analysis can be found in Table S1 in Multimedia Appendix 1. Variable transformations are detailed in Table S2 in Multimedia Appendix 1.

### Statistical Modelling With XGBoost

Python (v 3.8.13; Python Software Foundation) was used for all analyses. Three classification models were generated using the XGBoost algorithm [20], with the outcome being class labels derived from self-reported enthusiasm, modelled by our set of 50 input variables. XGBoost is a decision tree–based algorithm, whereby a set of trees are initialized and iterated during model training to improve fit. The weighted average of the output of each tree within the set is considered as the prediction of the trained model. XGBoost was selected due to its ability to handle linear, nonlinear, and interactive effects between predictor variables.

A total of 3 classification models were built, each for a different grouping of responses to our primary outcome of enthusiasm. These 3 models were specified as follows:

1. Multiclass classification: Outcome being the 4 classes of the original survey responses, that is, strongly disagree (470/13,661, 3.44%), somewhat disagree (1388/13,661, 10.16%), somewhat agree (6331/13,661, 46.34%), and strongly agree (5472/13,661, 40.06%) (N=13,661).
2. Binary classification: Outcome being binary with the 2 classes being students who chose strongly agree (5472/13,661, 40.06%) and students who chose any other response (8189/13,661, 59.94%) (N=13,661).
3. Binary classification: Outcome being binary with the first class being “enthusiastic” (5472/7330, 74.7%) comprising students who selected “strongly agree” and the second class being “not enthusiastic” (1858/7330, 25.3%) comprising students who chose either “strongly disagree” or “somewhat disagree.” Students who chose “somewhat agree” were removed from this analysis to improve class discrimination, as it was considered more likely to represent ambivalence when compared to a “somewhat disagree” response due to acquiescence bias [29]. Those who responded “somewhat disagree” were included to compensate for the low percentage of students in the “strongly disagree” class (n=7330).

In the binary classification of model 3, a sensitivity analysis to demonstrate ambivalence is reported in Multimedia Appendix 1. The data were divided into 2 nonoverlapping subsets through random sampling, stratified by outcome categories (to maintain balance in outcome groups), with 80% as training data and 20% as withheld test data to evaluate unbiased model performance. Random oversampling of the outcome group with fewer observations—within only the training data—was used to help mitigate distributional imbalances (using the imbalanced-learn Python library). The area under the receiver operating characteristic curve (AUROC) was used to evaluate the model’s accuracy on the validation data during the fitting process due to its scale and classification-threshold invariance. XGBoost automatically handled missing data by assignment of a default direction at each decision tree node such that loss of AUROC was minimized. The optimal hyperparameters for the XGBoost algorithm, which are values that control the XGBoost process and are selected before model training, were selected through Bayesian optimization using the Hyperopt Python library [30]. Hyperparameters that were optimized can be seen in Table S3 in Multimedia Appendix 1. Accuracy, precision, and recall were also calculated for each model within test data, and confusion matrices were used to visualize model performance.

### Determination of Variable Importance With SHAP

Given that XGBoost is not inherently interpretable, the importance of each input feature in model classification was determined by calculating the absolute mean SHAP values [21] based on the test data. A positive SHAP value indicated that the variable had a positive influence on the outcome (pushing the model classification toward enthusiastic), with a greater magnitude indicative of a greater impact on the model output. In addition, following our previously published approach [22], SHAP values for the interactions among the top 15 input features were calculated to determine the importance of interaction among these variables on classification in comparison to the importance of the individual variables. SHAP analysis was performed on all models; however, the best model based on the aforementioned metrics was selected to present detailed results and structure discussion.

### Ethics Approval and Recruitment

The 2019 OSDUHS was approved by the institutional review boards at the Center for Addiction and Mental Health, York University, and 34 Ontario school boards. Schools were randomly selected. After seeking approval from relevant school boards, the randomly selected schools were invited to participate in the survey. Schools that could not participate were replaced by schools within the same stratum. Once a school was approved, 1 or 2 classes in the relevant grades were randomly selected from a master list of all classes. Students in the selected classes were given a parental consent–student assent form to take home to parents, which explained the survey’s purpose and method. Only students with parental consent could participate. Students completed the survey in the classroom during regular school hours. The survey data were anonymous [24]. Generative artificial intelligence was not used in the creation of this manuscript. Analyses of OSDUHS data were approved by the CAMH Research Ethics Board (CAMH 099/2019).

## Results

### Participant Characteristics

Table 1 summarizes the demographic characteristics of the survey participants included in our study. The sample had a mean age of 14.9 (SD 1.8) years, was well balanced for biological sex assigned at birth (7612/13,661, 55.72% female), and included a majority of individuals who self-identified as White (8617/13,661, 63.08%).

**Table 1:** Baseline characteristics of the respondents used in the modelling process (N=13,661).

Characteristics		Values^a^
**Age (years)**		
	Mean (SD)	14.86 (1.77)
	Range (min-max)	9 (11-20)
	Missing responses (n)	5
**Grade**		
	Mean (SD)	9.61 (1.65)
	Range (min-max)	5 (7-12)
	Missing responses	10
**Sex at birth, n (%)**		
	Female	7612 (55.72)
	Male	6049 (44.28)
**Region, n (%)**		
	Greater Toronto Area (GTA)	5257 (38.48)
	Northern Ontario	918 (6.72)
	Western Ontario	4382 (32.08)
	Eastern Ontario	3104 (22.72)
**Race/ethnicity (respondents were allowed to select more than one), n (%)**		
	White	8617 (63.08)
	Chinese	766 (5.61)
	South Asian	1265 (9.26)
	Black	1293 (9.46)
	Indigenous	378 (2.77)
	Filipino	736 (5.39)
	Latin/Central/South American	584 (4.27)
	Southeast Asian	259 (1.90)
	West Asian/Arab	731 (5.35)
	Korean	124 (0.91)
	Japanese	64 (0.47)
	Missing responses	67 (0.49)

^a^The percentages shown are not weighted.

### XGBoost Modelling of Youth Enthusiasm

**Figure 1:**
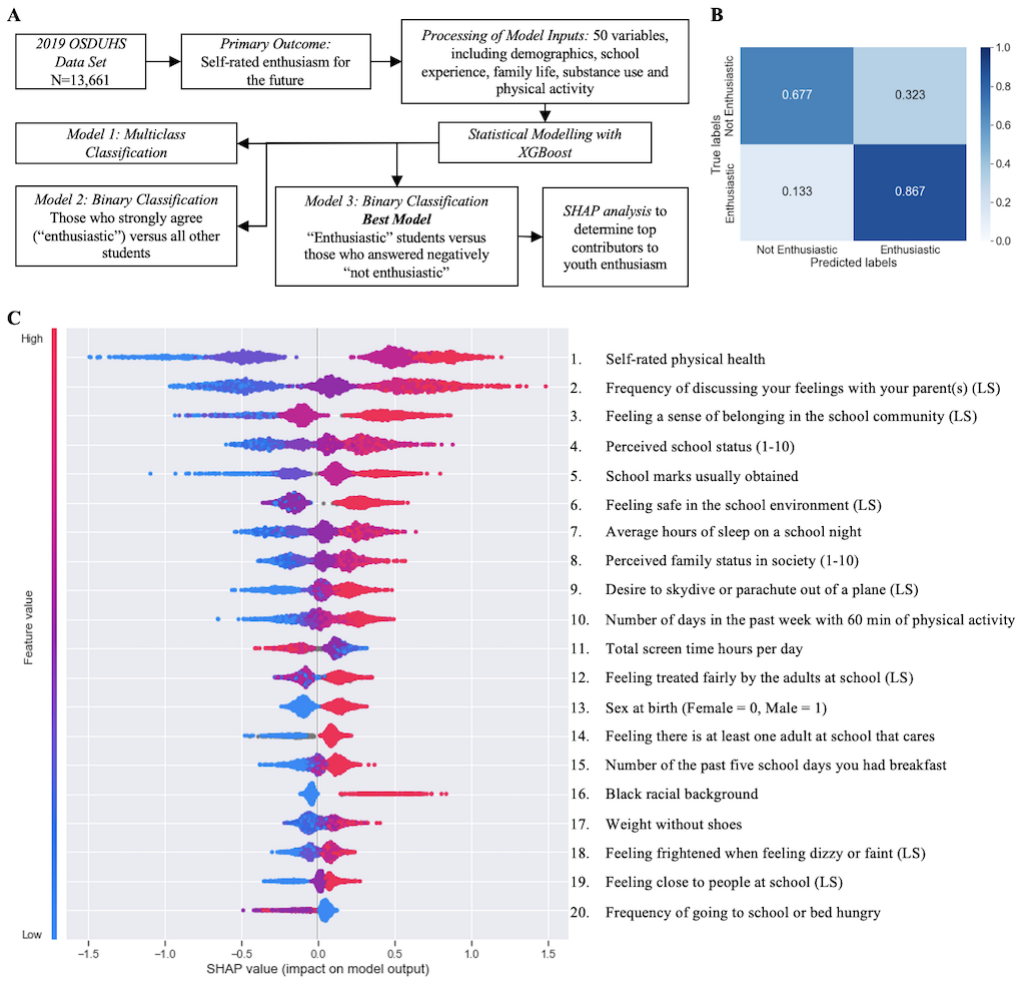
Results of the extreme gradient boosting model of binarized self-reported enthusiasm (model 3, n=7330). A: Simplified flow diagram of the study design. B: Confusion matrix for model 3, with elements normalized to the true prediction class population sizes. The main diagonal cells indicate predictions that match the true labels. C: Shapley additive explanations summary plot for model 3 showing the top 20 variables ranked by mean absolute Shapley additive explanations values on test data. Each point represents an individual student’s response to the question listed, the color of that point represents the actual value of the response, and the horizontal position of the point on the figure represents the impact that answer had on the predicted outcome. The further right on the figure the point is (indicating a higher SHAP value), the more positive impact it had toward predicting “enthusiastic,” as opposed to “not enthusiastic.” LS: Likert scale; OSDUHS: Ontario Student Drug Use and Health Survey; SHAP: Shapley additive explanations; XGBoost: extreme gradient boosting.

Table 2 summarizes the key performance metrics for the 3 models built using XGBoost: the multiclass classification of all enthusiasm responses (model 1), the binary classification of enthusiastic respondents against all others (model 2), and the binary classification of enthusiastic versus not enthusiastic respondents, excluding ambivalent classes (model 3). For each of the 3 models, the accuracy, precision, and recall metrics were highly similar, being within 1% of each other, indicating that the model classified a similar number of false positives as false negatives [31]. The AUROC metrics were 0.68 for model 1, 0.75 for model 2, and 0.86 for model 3. The AUROC metrics are shown in Table 2, which highlights the calculated performance metrics for each model on the withheld test data. Model 3 was selected as the best classification model for self-reported enthusiasm using the set of 50 input features, as it had the best performance across accuracy (0.81, 95% CI 0.79-0.83), precision (0.82, 95% CI 0.80-0.84), and recall (0.81, 95% CI 0.79-0.83) in held-out test data. The confusion matrix depicting model 3 performance is shown in Figure 1B. Confusion matrices for models 1 and 2 are available in Figures S1-S2 in Multimedia Appendix 1. Optimal hyperparameters for our trained model are provided in Table S3 in Multimedia Appendix 1. The superior performance of this model when compared to those of model 1 (accuracy 0.53, 95% CI 0.51-0.55) and model 2 (accuracy 0.68, 95% CI 0.66-0.70) is likely due to the exclusion of potentially ambivalent respondents, polarizing the sample and offering more contrast between classes for training.

**Table 2:** Performance metrics calculated for each model on withheld test data.

	Training data (80% of the data), n	Test data (20% of the data), n	AUROC^a^	Accuracy (95% CI)	Precision (95% CI)	Recall (95% CI)
Model 1: multiclass classification	10,888	2773	0.68	0.53 (0.51-0.55)	0.52 (0.50-0.54)	0.53 (0.51-0.55)
Model 2: binary classification (enthusiastic vs all others)	10,888	2773	0.75	0.68 (0.66-0.70)	0.69 (0.67-0.71)	0.68 (0.66-0.70)
Model 3: binary classification (enthusiastic vs not enthusiastic)	5864	1466	0.86	0.81 (0.79-0.83)	0.82 (0.80-0.84)	0.81 (0.79-0.83)

^a^AUROC: area under the receiver operating characteristic curve.

### Identifying the Most Important Variables With SHAP

**Figure 2:**
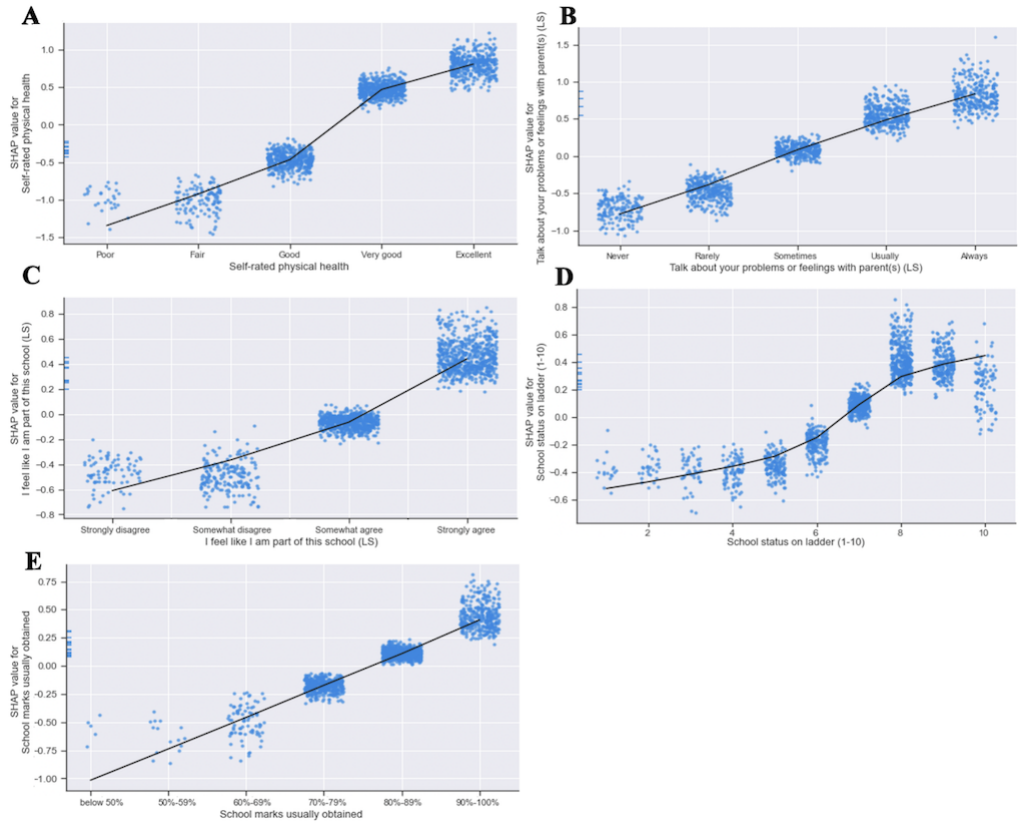
Shapley additive explanations values plotted for the top 5 variables predictive of binarized enthusiasm (model 3, n=7330). Each point represents a single student’s response to the question plotted against its corresponding Shapley additive explanations value (impact it had on predicting enthusiastic over not enthusiastic). Positive Shapley additive explanations values indicate more impact toward “enthusiastic,” whereas negative Shapley additive explanations values indicate more impact toward “not enthusiastic.” A line of best fit (locally weighted scatterplot smoothing smooth curve) was added to each plot only to demonstrate the overall trend; however, they were not fit using the underlying model and are not meant to represent statistical significance. A: Self-rated physical health (most impactful feature). B: “Talk about your problems or feelings with parent(s) (Likert scale)” (second most impactful feature). C: “I feel like I am part of this school (Likert scale)” (third most impactful feature on model prediction). D: School status on a scale of 1-10 (fourth most impactful feature). E: School marks usually obtained (fifth most impactful feature). Blue dashes on the y-axis indicate observations for which the variable indicated on the x-axis were missing. LS: Likert scale; SHAP: Shapley additive explanations.

SHAP analysis ranked the most important features used by the model for classification. Figure 1B shows the top 20 input variables, as determined by the mean of the absolute values of their SHAP values in the test data. Figure 2 illustrates the impact of the response of each individual student on model outputs for the top 5 input features within the test data. The most important contributor to youth enthusiasm based on SHAP analysis was self-rated physical health. In general, themes surrounding physical health, family relationships, and school experience appeared among the top input features across all 3 models. Details for the SHAP values of the top 5 input features in each model are listed in Table 3. A complete list of SHAP values for model 3 is available in Table S4 in Multimedia Appendix 1.

**Table 3:** Top 5 variables identified by magnitude of importance (rank) for all 3 models.

Rank	Model 1: multiclass classification		Model 2: binary classification (enthusiastic vs all others)		Model 3: binary classification (enthusiastic vs not enthusiastic)	
	Input feature	Mean absolute SHAP^a^ value	Input feature	Mean absolute SHAP value	Input feature	Mean absolute SHAP value
1	Self-rated physical health	0.299	Feeling comfortable sharing one’s thoughts or feelings with their parents	0.266	Self-rated physical health	0.621
2	Feeling comfortable sharing one’s thoughts or feelings with their parents	0.222	Self-rated physical health	0.245	Feeling comfortable sharing one’s thoughts or feelings with their parents	0.494
3	Feeling a sense of belonging in the school community	0.179	School marks usually obtained	0.203	Feeling a sense of belonging in the school community	0.322
4	Perceived school status on a scale of 1-10	0.173	Feeling a sense of belonging in the school community	0.164	Perceived school status on a scale of 1-10	0.260
5	Perceived family status in society on a scale of 1-10	0.126	Perceived school status on a scale of 1-10	0.137	School marks usually obtained	0.231

^a^SHAP: Shapley additive explanations.

### Assessment of Variable Interactions

Examination of the impact of pairwise variable interactions on model classification indicated that the interactions between input features did not substantially affect the model output, with mean absolute SHAP values for these interactions being lower than the mean absolute SHAP values of any of the top 15 most important explanatory variables in each of the 3 tested models. For our top model (model 3), the most important interaction with a SHAP value of 0.054 was between “talk about your problems or feelings with your parents” and “self-rated physical health.” Variable interaction data are presented in Figures S3-S8 in Multimedia Appendix 1.

## Discussion

We used a gradient-boosted tree-based machine learning algorithm to classify self-reported youth enthusiasm (as an indicator of well-being [14,15]) and identify the most important contributing input features by using the population-level OSDUHS, conducted among elementary and secondary school students attending publicly funded Ontario schools. A wide range of variables were used to model enthusiasm, including sociodemographic factors, physical activity and quantity of sleep, other physical and mental health indicators, perception of school experience, and substance use. The XGBoost algorithm was used to generate 3 models to classify youth enthusiasm, with SHAP values being used to explain the importance of each input feature across the sample. The top explanatory variables in classifying enthusiasm were related to physical health, relationship with parents, and school experience. A crucial aspect of this insight is that these rankings are derived from an approach that accounts for the context of all variables in the model (ie, the coalition of variables for each participant) in a nonlinear interactive way rather than in mass bivariate testing or specific hypothesis-driven tests.

Our ranking of top features supports a body of evidence describing the close interconnection between a person’s physical and mental health—both actual and perceived [32-34]. For example, our top feature contributing to enthusiasm and by proxy well-being was self-rated physical health, which has many contributors, including self-esteem, self-awareness, and physical activity levels. Physical activity itself is a well-known protective factor against mental illness [35-37], with physical activity in youth consistently improving well-being [34,38]. Conversely, physical inactivity is a potential risk factor for mental illness, with increasing physical activity contributing to more effective treatment [37]. Actual physical activity and self-perceived physical health status are related, and both could be used to identify children at risk for associated physical and mental health problems [32]. The number of days in the past week with 60 minutes of physical activity ranked tenth in variable importance. In addition to perceived and actual physical activity, number of hours slept per night was the seventh most important feature for classifying enthusiasm, with students who slept more hours reporting greater enthusiasm and therefore potentially greater sense of well-being. Previous studies have demonstrated a significant association between poor sleep quality and poor mental health. It is also possible that sleep is a mediator of other factors that contribute to poor mental health in youth, including social media use [39]. Given our findings suggesting a connection between physical health and enthusiasm, as well as the body of evidence demonstrating the interconnection of mental and physical health, it is likely that the promotion of physical health can enhance youth well-being [36]. Thus, public health measures directed at improving youth well-being should include the promotion of physical health and importantly, a healthy self-perception of physical fitness.

Relationships with parents and school, specifically as they relate to feeling socially supported in these environments, were also important predictors of enthusiasm, and therefore probably well-being (feeling comfortable sharing thoughts and feelings with parents, feeling a sense of belonging in the school community, feeling safe in school, and high perceived school status). The connection of family and school relationships with youth well-being has also been highlighted in several previous studies: family and teacher relationships have been demonstrated to be significantly associated with reduced substance use [40]. Peer connectedness in the school environment has also played a role with a greater sense of well-being [40]. Support from teachers and family has also been associated with significant improvements in mental well-being [41]. Additionally, it has been suggested that school environments that are structured to maximize connection between teachers and students can enhance student engagement and lead to improved student well-being [42,43]. The importance of social connectedness as it relates to youth enthusiasm may be even more relevant post COVID-19 pandemic. Although our study was conducted before the pandemic, a recent study of Canadian youth subjective well-being (an all-encompassing term for happiness, satisfaction, morale, and positive affect [44]) during the COVID-19 pandemic showed that having access to friends and areas to play was correlated with improved subjective well-being [10]. Given the connection of support within school and family environments with enthusiasm and youth well-being, increased social support should be another consideration in public health measures aimed at improving well-being in youth.

Our study has several limitations. First, our analyses were limited to the set of 50 core questions used in OSDUHS and excluded potentially important questions and topics unique to particular split ballot versions of the questionnaire. These topics included bullying, gambling, and antisocial behaviors that could also be related to enthusiasm. Second, the possible response categories for the self-reported enthusiasm for the future question in OSDUHS did not include a neutral option. Although we believe modelling the outcome in multiple ways helped provide a more fulsome picture of enthusiasm based on different groupings, we cannot assume that all respondents in the “somewhat agree” or “somewhat disagree” categories were truly ambivalent. Third, students who did not respond to the question of enthusiasm were excluded from the analysis. It is possible that this missingness is nonrandom, with students who felt less enthusiastic about their future deciding to not participate in the survey or to respond to the question. However, missingness for this variable was low (481/14,142, 3.5% missing of the total sample). Additionally, the OSDUHS responses are self-reported, meaning that they could be affected by recall and memory. Finally, it is to be noted that the conducted analysis is cross-sectional and not causal.

In summary, we used XGBoost to identify the set of behavioral, environmental, and psychosocial factors related to self-reported enthusiasm for the future in a large sample of young students. The most important factors were perceived physical health, school social status and connectedness, and quality of parental relationships. These factors were found to have a stronger association with enthusiasm than many common intervention targets, including social media, drug, and alcohol use. With the close interconnection of enthusiasm and well-being, our findings suggest that a focus on physical health and school connectedness should be central to impactful public health programming aimed at improving the mental well-being of youth, particularly when it comes to improving enthusiasm for the future.

## Appendix

Multimedia Appendix 1 Supplementary data.

## Abbreviations

AUROC: area under the receiver operating characteristic curve
OSDUHS: Ontario Student Drug Use and Health Survey
SHAP: Shapley additive explanations
XGBoost: extreme gradient boosting

## Data Availability

The data set analyzed during this study is available upon reasonable request to the Center for Addiction and Mental Health at osduhs@camh.ca.
