# Supplementary material for: Identifying Psychosocial and Ecological Determinants of Enthusiasm In Youth: Integrative Cross-Sectional Analysis Using Machine Learning
Source: JMIR Public Health Surveill. 2024 Sep 12;10:e48705. doi: 10.2196/48705 (PMC11427878; doi:10.2196/48705)
Supplement: Multimedia Appendix 1 [file publichealth_v10i1e48705_app1.docx]

# Multimedia Appendix 1

**Table S1:** Complete list of descriptive variables used in model creation.

| Question | Question Options (if non-numeric) or  Characteristics of Distribution (if numeric) | Distribution |
| --- | --- | --- |
| Age | Mean | 14.86 |
|  | Range | 9 |
|  | Standard Deviation | 1.77 |
|  | Missing | 5 |
| Grade (self-reported) | Mean | 9.61 |
|  | Range | 5 |
|  | Standard Deviation | 1.65 |
|  | Missing | 10 |
| Sex at birth | Male | 55.72% |
|  | Female | 44.28% |
|  | Missing | 0.00% |
| Region (4 basic regions) | GTA | 38.48% |
|  | North | 6.72% |
|  | West | 32.08% |
|  | East | 22.72% |
|  | Missing | 0.00% |
| Years lived in Canada | 2 years or less | 3.43% |
|  | 3 to 5 years | 3.67% |
|  | 6 to 10 years | 4.94% |
|  | 11 or more years | 5.60% |
|  | All of my life | 82.16% |
|  | Missing | 0.20% |
| Language usually spoken at home | English | 64.67% |
|  | French | 1.54% |
|  | English and French | 4.99% |
|  | Multiple Languages | 28.46% |
|  | Missing | 0.34% |
| Living situation at home | I live in one home only | 85.71% |
|  | I split time between homes | 13.36% |
|  | Missing | 0.93% |
| Household composition (Select all that apply) | Birth Mother | 91.96% |
|  | Stepmother | 2.83% |
|  | Adoptive mother | 0.96% |
|  | Birth father | 74.61% |
|  | Stepfather | 7.53% |
|  | Adoptive father | 1.02% |
|  | Grandparent(s) | 10.86% |
|  | Adult relative(s) | 2.81% |
|  | Foster parent(s) | 0.45% |
|  | Brother/stepbrother | 51.20% |
|  | Sister/stepsister | 46.45% |
|  | Others | 3.22% |
|  | Live alone | 0.19% |
|  | Missing | 0.57% |
| Background (Select all that apply) | White | 63.08% |
|  | Chinese | 5.61% |
|  | South Asian | 9.26% |
|  | Black | 9.46% |
|  | Indigenous | 2.77% |
|  | Filipino | 5.39% |
|  | Latin/Central/South American | 4.27% |
|  | Southeast Asian | 1.90% |
|  | West Asian/Arab | 5.35% |
|  | Korean | 0.91% |
|  | Japanese | 0.47% |
|  | Missing | 0.49% |
| School marks usually obtained | Below 50% | 0.26% |
|  | 50%-59% | 1.09% |
|  | 60%-69% | 5.89% |
|  | 70%-79% | 30.60% |
|  | 80%-89% | 42.74% |
|  | 90%-100% | 18.74% |
|  | Missing | 0.68% |
| Enrolled in special education or have an individual education plan | No | 78.51% |
|  | Yes | 13.10% |
|  | Missing | 8.39% |
| Number of school days missed in last 4 weeks | Mean | 1.39 |
|  | Range | 20 |
|  | Standard Deviation | 2.20 |
|  | Missing | 402 |
| I feel safe in my school | Strongly disagree | 1.68% |
|  | Somewhat disagree | 6.64% |
|  | Somewhat agree | 47.49% |
|  | Strongly agree | 42.60% |
|  | Missing | 1.59% |
| I feel close to people at this school | Strongly disagree | 3.98% |
|  | Somewhat disagree | 10.09% |
|  | Somewhat agree | 44.81% |
|  | Strongly agree | 40.04% |
|  | Missing | 1.08% |
| I feel like I am part of this school | Strongly disagree | 4.72% |
|  | Somewhat disagree | 12.36% |
|  | Somewhat agree | 44.95% |
|  | Strongly agree | 36.94% |
|  | Missing | 1.02% |
| I am treated fairly by the adults at my school | Strongly disagree | 4.96% |
|  | Somewhat disagree | 11.18% |
|  | Somewhat agree | 39.48% |
|  | Strongly agree | 43.26% |
|  | Missing | 1.11% |
| Do you feel there is at least one adult at your school that cares and you could talk to if you needed help? | No | 9.87% |
|  | Yes | 72.68% |
|  | Missing | 17.44% |
| School status on ladder (1-10) | Mean | 6.81 |
|  | Range | 9 |
|  | Standard Deviation | 1.87 |
|  | Missing | 143 |
| Parents born in Canada | No parent born in Canada | 35.45% |
|  | One parent born in Canada | 13.16% |
|  | Two (or more) parents born in Canada | 49.73% |
|  | Missing | 1.65% |
| Father's highest level of education | Did not attend high school | 1.28% |
|  | Attended high school | 5.70% |
|  | Graduated high school | 10.56% |
|  | Attended college | 4.00% |
|  | Graduated college | 18.07% |
|  | Attended university | 2.55% |
|  | Graduated university | 35.12% |
|  | Missing | 22.71% |
| Mother's highest level of education | Did not attend high school | 1.11% |
|  | Attended high school | 3.20% |
|  | Graduated high school | 9.21% |
|  | Attended college | 3.63% |
|  | Graduated college | 21.49% |
|  | Attended university | 2.75% |
|  | Graduated university | 41.38% |
|  | Missing | 17.24% |
| Do you talk about your problems or feelings with parent(s)? | Never | 11.92% |
|  | Rarely | 23.16% |
|  | Sometimes | 24.72% |
|  | Usually | 24.73% |
|  | Always | 15.10% |
|  | Missing | 0.36% |
| Family status in society on ladder (1-10) | Mean | 6.98 |
|  | Range | 9 |
|  | Standard Deviation | 1.62 |
|  | Missing | 189 |
| Height without shoes | Mean | 166.75 |
|  | Range | 66 |
|  | Standard Deviation | 10.78 |
|  | Missing | 400 |
| Weight without shoes | Mean | 61.05 |
|  | Range | 91 |
|  | Standard Deviation | 15.88 |
|  | Missing | 516 |
| Self-rated physical health | Poor | 1.68% |
|  | Fair | 8.43% |
|  | Good | 29.84% |
|  | Very good | 37.59% |
|  | Excellent | 21.78% |
|  | Missing | 0.69% |
| Number of days in past 7 physically active for total 60 minutes per day | 0 days | 8.33% |
|  | 1 day | 6.57% |
|  | 2 days | 9.82% |
|  | 3 days | 12.96% |
|  | 4 days | 12.86% |
|  | 5 days | 17.74% |
|  | 6 days | 9.67% |
|  | 7 days | 21.23% |
|  | Missing | 0.81% |
| How many times do you eat fruits and vegetables on an average day? | 0 times a day | 2.73% |
|  | 1 time a day | 15.24% |
|  | 2 times a day | 26.79% |
|  | 3 times a day | 25.37% |
|  | 4 times a day | 13.97% |
|  | 5 times a day | 6.05% |
|  | 6 or more times a day | 8.39% |
|  | Missing | 1.46% |
| How often do you drink pop, sport drink, sweetened drinks last 7 days? | Did not drink any of these in last 7 days | 16.73% |
|  | 1 time in last 7 days | 23.65% |
|  | 2 to 4 times | 39.08% |
|  | 5 to 6 times | 8.80% |
|  | Once each day | 5.74% |
|  | More than once each day | 4.79% |
|  | Missing | 1.21% |
| How many of last 5 school days did you eat breakfast? | None | 23.83% |
|  | 1 to 2 days | 18.94% |
|  | 3 to 4 days | 13.77% |
|  | All 5 days | 42.24% |
|  | Missing | 1.22% |
| How often did you go to school or bed hungry? | Never | 74.56% |
|  | Sometimes | 18.52% |
|  | Often | 3.94% |
|  | Always | 1.63% |
|  | Missing | 1.35% |
| How many hours of sleep get on average school night? | 4 hours or less | 5.25% |
|  | 5 hours | 8.99% |
|  | 6 hours | 19.49% |
|  | 7 hours | 26.65% |
|  | 8 hours | 22.82% |
|  | 9 hours | 10.70% |
|  | 10 hours | 4.22% |
|  | 11 or more hours | 1.08% |
|  | Missing | 0.80% |
| Have you had a head injury that resulted in headache/dizziness/blurred vision/vomiting/unconsciousness in the last 12 months? | Never had head injury like this in my life | 60.18% |
|  | Had head injury like this, but not in last 12 months | 23.88% |
|  | Yes, had injury like this in last 12 months | 15.45% |
|  | Missing | 0.50% |
| Hours per day watching TV/movies, playing video games, on Internet in free time | None | 0.54% |
|  | Less than 1 hour a day | 3.88% |
|  | 1 to 2 hours a day | 20.96% |
|  | 3 to 4 hours a day | 35.98% |
|  | 5 to 6 hours a day | 20.01% |
|  | 7 or more hours a day | 13.31% |
|  | Missing | 5.33% |
| Hours on social media per day | Don't use social media | 7.51% |
|  | Use social media, but not daily | 5.64% |
|  | Less than 1 hour a day | 5.92% |
|  | About 1 hour a day | 12.17% |
|  | 2 hours a day | 20.46% |
|  | 3 to 4 hours a day | 27.85% |
|  | 5 to 6 hours a day | 13.91% |
|  | 7 or more hours a day | 6.28% |
|  | Missing | 0.26% |
| Have you ever posted something personal on social media and now regret it? | No | 78.98% |
|  | Yes | 20.54% |
|  | Missing | 0.48% |
| I would like to skydive or parachute out of a plane | Strongly disagree | 24.27% |
|  | Somewhat disagree | 14.85% |
|  | Somewhat agree | 26.48% |
|  | Strongly agree | 34.24% |
|  | Missing | 0.16% |
| I usually act without stopping to think | Strongly disagree | 15.06% |
|  | Somewhat disagree | 38.47% |
|  | Somewhat agree | 35.33% |
|  | Strongly agree | 10.78% |
|  | Missing | 0.36% |
| It frightens me when I feel dizzy or faint | Strongly disagree | 17.17% |
|  | Somewhat disagree | 27.57% |
|  | Somewhat agree | 36.15% |
|  | Strongly agree | 18.27% |
|  | Missing | 0.85% |
| How many times did you ride in vehicle driven by someone been drinking alcohol in the last 12 months? | Never | 76.57% |
|  | Once | 5.72% |
|  | 2 times | 2.96% |
|  | 3 times | 1.51% |
|  | 4 times | 0.89% |
|  | 5 times | 0.63% |
|  | 6 times | 0.34% |
|  | 7 times | 0.20% |
|  | 8 or more times | 1.63% |
|  | Missing | 9.55% |
| How many times did you ride in vehicle driven by someone been using drugs in the last 12 months? | Never | 83.58% |
|  | Once | 3.46% |
|  | 2 times | 1.76% |
|  | 3 times | 0.97% |
|  | 4 times | 0.57% |
|  | 5 times | 0.42% |
|  | 6 times | 0.23% |
|  | 7 times | 0.12% |
|  | 8 or more times | 2.08% |
|  | Missing | 6.82% |
| Smoked tobacco cigarettes in the past 12 months | No/a few puffs | 95.33% |
|  | Yes | 4.38% |
|  | Missing | 0.29% |
| Smoked 1+ tobacco cigarette daily in the past 12 months | No/a few puffs | 98.39% |
|  | Yes | 1.32% |
|  | Missing | 0.29% |
| Drank alcohol in the past 12 months | No/a sip | 59.31% |
|  | Yes | 39.84% |
|  | Missing | 0.86% |
| Had 5+ drinks at least once past 4 weeks (binge drinking) | No | 85.56% |
|  | Yes | 13.99% |
|  | Missing | 0.45% |
| Used cannabis (any) past 12 months | No | 78.90% |
|  | Yes | 20.43% |
|  | Missing | 0.67% |
| Used cough/cold medication (dextromethorphan) to 'get high' in the past 12 months | No | 92.39% |
|  | Yes | 7.00% |
|  | Missing | 0.61% |
| Sniffed inhalants (glue/solvent) in the past 12 months | No | 96.52% |
|  | Yes | 3.05% |
|  | Missing | 0.42% |
| Used prescription opioid pain reliever non-medically in the past 12 months | No | 88.95% |
|  | Yes | 9.88% |
|  | Missing | 1.16% |
| Used ADHD drug non-medically in the past 12 months | No | 96.57% |
|  | Yes | 2.34% |
|  | Missing | 1.08% |
| Primary Outcome: I am very enthusiastic about my future | Strongly disagree | 3.44% |
|  | Somewhat disagree | 10.16% |
|  | Somewhat agree | 46.34% |
|  | Strongly agree | 40.06% |

**Table S2:** Transformations of original variables for use in XGBoost models.

| **Question** | **Original** | **Transformation** |
| --- | --- | --- |
| Age | Numeric | Numeric |
| Grade (self-reported) | Numeric | Numeric |
| Sex at birth | Male, Female | Male, Female |
| Region (4 basic regions) | GTA, North, West, East | GTA, North, West, East  One Hot Encoded |
| Years lived in Canada | All of my life' < '2 years or less' < '3 to 5 years' < '6 to 10 years' < '11 or more years' | 2 years or less' < '3 to 5 years' < '6 to 10 years' < '11 or more years' < 'All of my life' |
| Language usually spoken at home | English', 'French', 'English and French', 'English, French and another language', 'English and another language', 'French and another language', 'Other languages', 'Other combinations' | English', 'French', 'English and French', 'Other/Multiple Languages'  One Hot Encoded |
| Living situation at home | I live in one home only', 'I split time between homes' | I live in one home only', 'I split time between homes' |
| Household composition | Birth Mother', 'Stepmother', 'Adoptive mother', 'Birth father', 'Stepfather', 'Adoptive father', 'Grandparent(s)', 'Adult relative(s)', 'Foster parent(s)', 'Brother/stepbrother', 'Sister/stepsister', 'Others', 'Live alone' | Birth Mother', 'Stepmother', 'Adoptive mother', 'Birth father', 'Stepfather', 'Adoptive father', 'Grandparent(s)', 'Adult relative(s)', 'Foster parent(s)', 'Brother/stepbrother', 'Sister/stepsister', 'Others', 'Live alone'  One Hot Encoded |
| Background (Select all that apply) | White', 'Chinese', 'South Asian', 'Black', 'Indigenous', 'Filipino', 'Latin/Central/South American', 'Southeast Asian', 'West Asian/Arab', 'Korean', 'Japanese', 'Unsure' | White', 'Chinese', 'South Asian', 'Black', 'Indigenous', 'Filipino', 'Latin/Central/South American', 'Southeast Asian', 'West Asian/Arab', 'Korean', 'Japanese'  'Unsure' was consisdered missing. One Hot Encoded |
| School marks usually obtained | 90%-100%' < '80%-89%' < '70%-79%' < '60%-69%' < '50%-59%' < 'below 50%' | below 50%' < '50%-59%' < '60%-69%' < '70%-79%' < '80%-89%' < '90%-100%' |
| Enrolled in special education or have an individual education plan | Yes', 'No', 'Not sure' | Yes', 'No'  'Not sure' was considered missing. |
| Number of school days missed in last 4 weeks | Numeric | Numeric |
| I feel safe in my school | Strongly agree' < 'Somewhat agree' < 'Somewhat disagree' < 'Strongly disagree' | Strongly disagree' < 'Somewhat disagree' < 'Somewhat agree' < 'Strongly agree' |
| I feel close to people at this school | Strongly agree' < 'Somewhat agree' < 'Somewhat disagree' < 'Strongly disagree' | Strongly disagree' < 'Somewhat disagree' < 'Somewhat agree' < 'Strongly agree' |
| I feel like I am part of this school | Strongly agree' < 'Somewhat agree' < 'Somewhat disagree' < 'Strongly disagree' | Strongly disagree' < 'Somewhat disagree' < 'Somewhat agree' < 'Strongly agree' |
| I am treated fairly by the adults at my school | Strongly agree' < 'Somewhat agree' < 'Somewhat disagree' < 'Strongly disagree' | Strongly disagree' < 'Somewhat disagree' < 'Somewhat agree' < 'Strongly agree' |
| Do you feel there is at least one adult at your school that cares and you could talk to if you needed help? | Yes', 'No', 'Not sure' | Yes', 'No'  'Not sure' was considered missing. |
| School status on ladder (1-10) | Lowest standing', 2.0, 3.0, 4.0, 5.0, 6.0, 7.0, 8.0, 9.0, 'Highest standing' | Numeric scale 1-10 |
| Parents born in Canada | Two (or more) parents born in Canada' < 'One parent born in Canada' < 'No parent born in Canada' | No parent born in Canada' < 'One parent born in Canada' < 'Two (or more) parents born in Canada' |
| Father's highest level of education | Graduated university' < 'Attended university' < 'Graduated college' < 'Attended college' < 'Graduated high school' < 'Attended high school' < 'Did not attend high school' < 'Don't know' < 'No father' | Did not attend high school' < 'Attended high school' < 'Graduated high school' < 'Attended college' < 'Graduated college' < 'Attended university' < 'Graduated university'  'Don't know' and 'No father' were considered missing. |
| Mother's highest level of education | Graduated university' < 'Attended university' < 'Graduated college' < 'Attended college' < 'Graduated high school' < 'Attended high school' < 'Did not attend high school' < 'Don't know' < 'No mother' | Did not attend high school' < 'Attended high school' < 'Graduated high school' < 'Attended college' < 'Graduated college' < 'Attended university' < 'Graduated university'  'Don't know' and 'No mother' were considered missing. |
| Do you talk about your problems or feelings with parent(s)? | Always' < 'Usually' < 'Sometimes' < 'Rarely' < 'Never' | Never' < 'Rarely' < 'Sometimes' < 'Usually' < 'Always' |
| Family status in society on ladder (1-10) | Worst off', 2.0, 3.0, 4.0, 5.0, 6.0, 7.0, 8.0, 9.0, 'Best off' | Numeric scale 1 - 10 |
| Height without shoes | 4 feet 4 inches/ 132 cm or less',  '4 feet 5 inches/ 135 cm',  '4 feet 6 inches/ 137 cm',  '4 feet 7 inches/ 140 cm',  '4 feet 8 inches/ 142 cm',  '4 feet 9 inches/ 145 cm',  '4 feet 10 inches/147 cm',  '4 feet 11 inches/150 cm',  '5 feet 0 inches/ 152 cm',  '5 feet 1 inch/ 155 cm',  '5 feet 2 inches/ 157 cm',  '5 feet 3 inches/ 160 cm',  '5 feet 4 inches/ 163 cm',  '5 feet 5 inches/ 165 cm',  '5 feet 6 inches/ 168 cm',  '5 feet 7 inches/ 170 cm',  '5 feet 8 inches / 173 cm',  '5 feet 9 inches/ 175 cm',  '5 feet 10 inches/ 178 cm',  '5 feet 11 inches/ 180 cm',  '6 feet 0 inches / 183 cm',  '6 feet 1 inch/ 185 cm',  '6 feet 2 inches/ 188 cm',  '6 feet 3 inches/ 191 cm',  '6 feet 4 inches/ 193 cm',  '6 feet 5 inches/ 196 cm',  '6 feet 6 inches/ 198 cm or more' | Converted to the integer equivalent, for instance: 132, 135, 137, 140, etc. |
| Weight without shoes | 80 pounds/ 36 kg or less',  '81-85 pounds/ 37-39 kg',  '86-90 pounds/ 39-41 kg',  '91-95 pounds/ 41-43 kg',  '96-100 pounds/ 43-45 kg',  '101-105 pounds/ 46-48 kg',  '106-110 pounds/ 48-50 kg',  '111-115 pounds/ 50-52 kg',  '116-120 pounds/ 53-54 kg',  '121-125 pounds/ 55-57 kg',  '126-130 pounds/ 57-59 kg',  '131-135 pounds/ 59-61 kg',  '136-140 pounds/ 62-64 kg',  '141-145 pounds/ 64-66 kg',  '146-150 pounds/ 66-68 kg',  '151-155 pounds/ 68-70 kg',  '156-160 pounds/ 71-73 kg',  '161-165 pounds/ 73-75 kg',  '166-170 pounds/ 75-77 kg',  '171-175 pounds/ 77-79 kg',  '176-180 pounds/ 80-82 kg',  '181-185 pounds/ 82-84 kg',  '186-190 pounds/ 84-86 kg',  '191-195 pounds/ 87-88 kg',  '196-200 pounds/ 89-91 kg',  '201-205 pounds/ 91-93 kg',  '206-210 pounds/ 93-95 kg',  '211-215 pounds/ 96-98 kg',  '216-220 pounds/ 98-100 kg',  '221-225 pounds/100-102 kg',  '226-230 pounds/102-104 kg',  '231-235 pounds/105-107 kg',  '236-240 pounds/107-109 kg',  '241-245 pounds/109-111 kg',  '246-250 pounds/112-114 kg',  '251-255 pounds/114-116 kg',  '256-260 pounds/116-118 kg',  '261-265 pounds/118-120 kg',  '266-270 pounds/121-122 kg',  '271-275 pounds/123-125 kg',  '276-280 pounds/125-127 kg',  '281 pounds/127 kg or more' | Converted to an integer scale by using the average of the 2kg range options, for instance: 36.0, 38.0, 40.0, 42.0, etc. |
| Self-rated physical health | Excellent' < 'Very good' < 'Good' < 'Fair' < 'Poor' | Poor' < 'Fair' < 'Good' < 'Very good' < 'Excellent' |
| Number of days in past 7 physically active for total 60 minutes per day | 0 days' < '1 day' < '2 days' < '3 days' < '4 days' < '5 days' < '6 days' < '7 days' | 0 days' < '1 day' < '2 days' < '3 days' < '4 days' < '5 days' < '6 days' < '7 days' |
| How many times do you eat fruits and vegetables on an average day? | 0 times a day' < '1 time a day' < '2 times a day' < '3 times a day' < '4 times a day' < '5 times a day' < '6 or more times a day' | 1 times a day' < '1 time a day' < '2 times a day' < '3 times a day' < '4 times a day' < '5 times a day' < '6 or more times a day' |
| How often do you drink pop, sport drink, sweetened drinks last 7 days? | 1 time in last 7 days' < '2 to 4 times' < '5 to 6 times' < 'Once each day' < 'More than once each day' < 'Did not drink any of these in last 7 days' | Did not drink any of these in last 7 days' < '1 time in last 7 days' < '2 to 4 times' < '5 to 6 times' < 'Once each day' < 'More than once each day' |
| How many of last 5 school days did you eat breakfast? | None' < '1 to 2 days' < '3 to 4 days' < 'All 5 days' | None' < '1 to 2 days' < '3 to 4 days' < 'All 5 days' |
| How often did you go to school or bed hungry? | Always', 'Often', 'Sometimes', 'Never' | Never' < 'Sometimes' < 'Often' < 'Always' |
| How many hours of sleep get on average school night? | 4 hours or less' < '5 hours' < '6 hours' '7 hours' < '8 hours'< '9 hours' < '10 hours' < '11 or more hours' | 5 hours or less' < '5 hours' < '6 hours' '7 hours' < '8 hours'< '9 hours' < '10 hours' < '11 or more hours' |
| Have you had a head injury that resulted in headache/dizziness/blurred vision/vomiting/unconsciousness in the last 12 months? | Never had head injury like this in my life', 'Had head injury like this, but not in last 12 months', 'Yes, had injury like this in last 12 months' | Never had head injury like this in my life', 'Had head injury like this, but not in last 12 months', 'Yes, had injury like this in last 12 months' |
| Hours per day watching TV/movies, playing video games, on Internet in free time | None' < 'Less than 1 hour a day' < '1 to 2 hours a day' < '3 to 4 hours hours a day' < '5 to 6 hours hours a day' < '7 or more hours a day' < 'Not sure' | None' < 'Less than 1 hour a day' < '1 to 2 hours a day' < '3 to 4 hours hours a day' < '5 to 6 hours hours a day' < '7 or more hours a day'  'Not sure' was considered missing. |
| Hours on social media per day | Less than 1 hour a day' < 'About 1 hour a day' < '2 hours a day' < '3 to 4 hours a day' < '5 to 6 hours a day' < '7 or more hours a day' < 'Use social media, but not daily' < 'Use Internet, but not social media' < 'Don't use the Internet' | Don't use social media' < 'Use social media, but not daily' < 'Less than 1 hour a day' < 'About 1 hour a day' < '2 hours a day' < '3 to 4 hours a day' < '5 to 6 hours a day' < '7 or more hours a day'  'Use Internet, but not social media' and 'Don't use the Internet' were considered 'Don't use social media' |
| Have you ever posted something personal on social media and now regret it? | Don't use social media' < 'Yes' < 'No' | 'Yes', 'No'  'Don’t use social media' was considered 'No' |
| I would like to skydive or parachute out of a plane | Strongly agree' < 'Somewhat agree' < 'Somewhat disagree' < 'Strongly disagree' | Strongly disagree' < 'Somewhat disagree' < 'Somewhat agree' < 'Strongly agree' |
| I usually act without stopping to think | Strongly agree' < 'Somewhat agree' < 'Somewhat disagree' < 'Strongly disagree' | Strongly disagree' < 'Somewhat disagree' < 'Somewhat agree' < 'Strongly agree' |
| It frightens me when I feel dizzy or faint | Strongly agree' < 'Somewhat agree' < 'Somewhat disagree' < 'Strongly disagree' | Strongly disagree' < 'Somewhat disagree' < 'Somewhat agree' < 'Strongly agree' |
| How many times did you ride in vehicle driven by someone been drinking alcohol in the last 12 months? | Never' < 'Once' < '2 times' < '3 times' < '4 times' < '5 times' < '6 times' < '7 times' < '8 or more times' < 'Not sure' | Never' < 'Once' < '2 times' < '3 times' < '4 times' < '5 times' < '6 times' < '7 times' < '8 or more times'  'Not sure' was considered missing. |
| How many times did you ride in vehicle driven by someone been using drugs in the last 12 months? | Never' < 'Once' < '2 times' < '3 times' < '4 times' < '5 times' < '6 times' < '7 times' < '8 or more times' < 'Not sure' | Never' < 'Once' < '2 times' < '3 times' < '4 times' < '5 times' < '6 times' < '7 times' < '8 or more times'  'Not sure' was considered missing. |
| Smoked tobacco cigarettes in the past 12 months | no/few puffs', 'yes' | no/few puffs', 'yes' |
| Smoked 1+ tobacco cigarette daily in the past 12 months | no', 'yes' | no', 'yes' |
| Drank alcohol in the past 12 months | no (incl sip)', 'yes' | no (incl sip)', 'yes' |
| Had 5+ drinks at least once past 4 weeks (binge drinking) | no', 'yes' | no', 'yes' |
| Used cannabis (any) past 12 months | no', 'yes' | no', 'yes' |
| Used cough/cold medication (dextromethorphan) to 'get high' in the past 12 months | no', 'yes' | no', 'yes' |
| Sniffed inhalants (glue/solvent) in the past 12 months | no', 'yes' | no', 'yes' |
| Used prescription opioid pain reliever non-medically in the past 12 months | no', 'yes' | no', 'yes' |
| Used ADHD drug non-medically in the past 12 months | no', 'yes' | no', 'yes' |
| Primary Outcome: I am very enthusiastic about my future | Strongly agree' < 'Somewhat agree' < 'Somewhat disagree' < 'Strongly disagree' | Strongly disagree' < 'Somewhat disagree' < 'Somewhat agree' < 'Strongly agree' |

**Table S3:** Results of hyperparameter tuning for the three models.

| **Hyperparameter Label** | **Values** | | |
| --- | --- | --- | --- |
|  | **Model 1** | **Model 2** | **Model 3** |
| **Number of trees** | 114 | 121 | 138 |
| **Maximum depth** | 10 | 6 | 7 |
| **Gamma** | 0.56653 | 2.31712 | 0.74259 |
| **Minimum child weight** | 6 | 5 | 7 |
| **L1 regularization term** | 0 | 0 | 0 |
| **L2 regularization term** | 1 | 1 | 1 |
| **Column subsampling** | 0.72345 | 0.82105 | 0.85910 |
| **Learning rate** | 0.21859 | 0.11812 | 0.06278 |
| **Subsampling ratio** | 0.98387 | 0.82222 | 0.97982 |

**Table S4:** All SHAP values for Model 3 ranked by order of magnitude. n_3_ = 7330. (LS = Likert Scale).

|  | **Input Feature** | **Mean Absolute SHAP Value** |
| --- | --- | --- |
| **1** | Self-rated physical health | 0.621 |
| **2** | Talk about your problems or feelings with parent(s) (LS) | 0.494 |
| **3** | I feel like I am part of this school (LS) | 0.322 |
| **4** | School status on ladder (1-10) | 0.260 |
| **5** | School marks usually obtained | 0.231 |
| **6** | I feel safe in my school (LS) | 0.222 |
| **7** | How many hours of sleep get on average school night? | 0.212 |
| **8** | Family status in society on ladder (1-10) | 0.167 |
| **9** | I would like to skydive or parachute out of a plane | 0.156 |
| **10** | Number of days in past 7 physically active for total 60 minutes per day | 0.153 |
| **11** | Hours per day watching TV/movies, playing video games, on internet in free time | 0.136 |
| **12** | I am treated fairly by the adults at my school (LS) | 0.123 |
| **13** | Sex at birth (Male = 1, Female = 0) | 0.122 |
| **14** | Do you feel there is at least one adult at your school that cares about you? | 0.112 |
| **15** | How many of last 5 school days did you eat breakfast? | 0.099 |
| **16** | Black: background | 0.094 |
| **17** | Weight without shoes | 0.083 |
| **18** | It frightens me when I feel dizzy or faint (LS) | 0.082 |
| **19** | I feel close to people at this school (LS) | 0.073 |
| **20** | How often go to school or bed hungry? | 0.070 |
| **21** | Chinese: background | 0.066 |
| **22** | Mother's highest level of education | 0.063 |
| **23** | Used cannabis (any) past 12 months? (all forms) | 0.059 |
| **24** | How many times eat fruits and vegetables on an average day? | 0.057 |
| **25** | I usually act without stopping to think (LS) | 0.057 |
| **26** | Height without shoes | 0.056 |
| **27** | Number of school days missed in last 4 weeks | 0.052 |
| **28** | Grade (self-reported) | 0.051 |
| **29** | Enrolled in special education/individual education plan | 0.049 |
| **30** | How many times did you ride in vehicle driven by someone drinking alcohol 12m? | 0.043 |
| **31** | Region: East | 0.040 |
| **32** | Father's highest level of education | 0.038 |
| **33** | Hours on social media per day | 0.037 |
| **34** | Sister/step: household composition | 0.035 |
| **35** | Age | 0.031 |
| **36** | Head injury that resulted in headache/dizziness/blurred vision/vomiting/unconsciousness/etc.? | 0.031 |
| **37** | Region: West | 0.029 |
| **38** | Drank alcohol in the past 12 months? (excludes a sip (all forms)) | 0.028 |
| **39** | Ever posted something personal on social media, and now regret it? | 0.027 |
| **40** | How often drink pop, sport drink, sweetened drinks last 7 days? | 0.026 |
| **41** | Were your parents born in Canada? | 0.025 |
| **42** | How many times did you ride in vehicle driven by someone using drugs in the last 12 mos? | 0.023 |
| **43** | Region: GTA | 0.023 |
| **44** | Birth father: household composition | 0.022 |
| **45** | Multiple Languages: Language at home | 0.022 |
| **46** | English and French: Language at home | 0.022 |
| **47** | English: Language at home | 0.021 |
| **48** | Used prescription opioid pain reliever NONmedically in the past 12 mos? (all forms) | 0.020 |
| **49** | Used ADHD drug NONmedically in the past 12 mos? (all forms) | 0.017 |
| **50** | Brother/step: household composition | 0.017 |
| **51** | White: background | 0.016 |
| **52** | Korean: background | 0.012 |
| **53** | Years lived in Canada | 0.011 |
| **54** | Filipino: background | 0.011 |
| **55** | South Asian: background | 0.011 |
| **56** | Birth mother: household composition | 0.010 |
| **57** | Sniffed inhalants (glue/solvent) in the past 12 mos? (all forms) | 0.010 |
| **58** | Living situation at home | 0.008 |
| **59** | Had 5+ drinks at least once in the past 4 weeks (binge drinking)? (all forms) | 0.007 |
| **60** | Stepmother: household composition | 0.006 |
| **61** | Grandparent(s): household composition | 0.006 |
| **62** | Others: household composition | 0.006 |
| **63** | Region: North | 0.005 |
| **64** | West Asian/Arab: background | 0.005 |
| **65** | Used cough/cold medication (dextromethorphan) to “get high” in the past 12 mos? (all forms) | 0.005 |
| **66** | Smoked tobacco cigarettes in the past 12 mos? (excludes a few puffs (all forms)) | 0.004 |
| **67** | Stepfather: household composition | 0.004 |
| **68** | Live Alone: household composition | 0.002 |
| **69** | Adult relative(s): household composition | 0.002 |
| **70** | Latin/Central/South American: background | 0.002 |
| **71** | Indigenous: background | 0.002 |
| **72** | French: Language at home | 0.000 |
| **73** | Southeast Asian: background | 0.000 |
| **74** | Smoked 1+ tobacco cigarette daily in the past 12 mos? (all forms) | 0.000 |
| **75** | Adoptive mother: household composition | 0.000 |
| **76** | Adoptive father: household composition | 0.000 |
| **77** | Foster parent(s): household composition | 0.000 |
| **78** | Japanese: background | 0.000 |


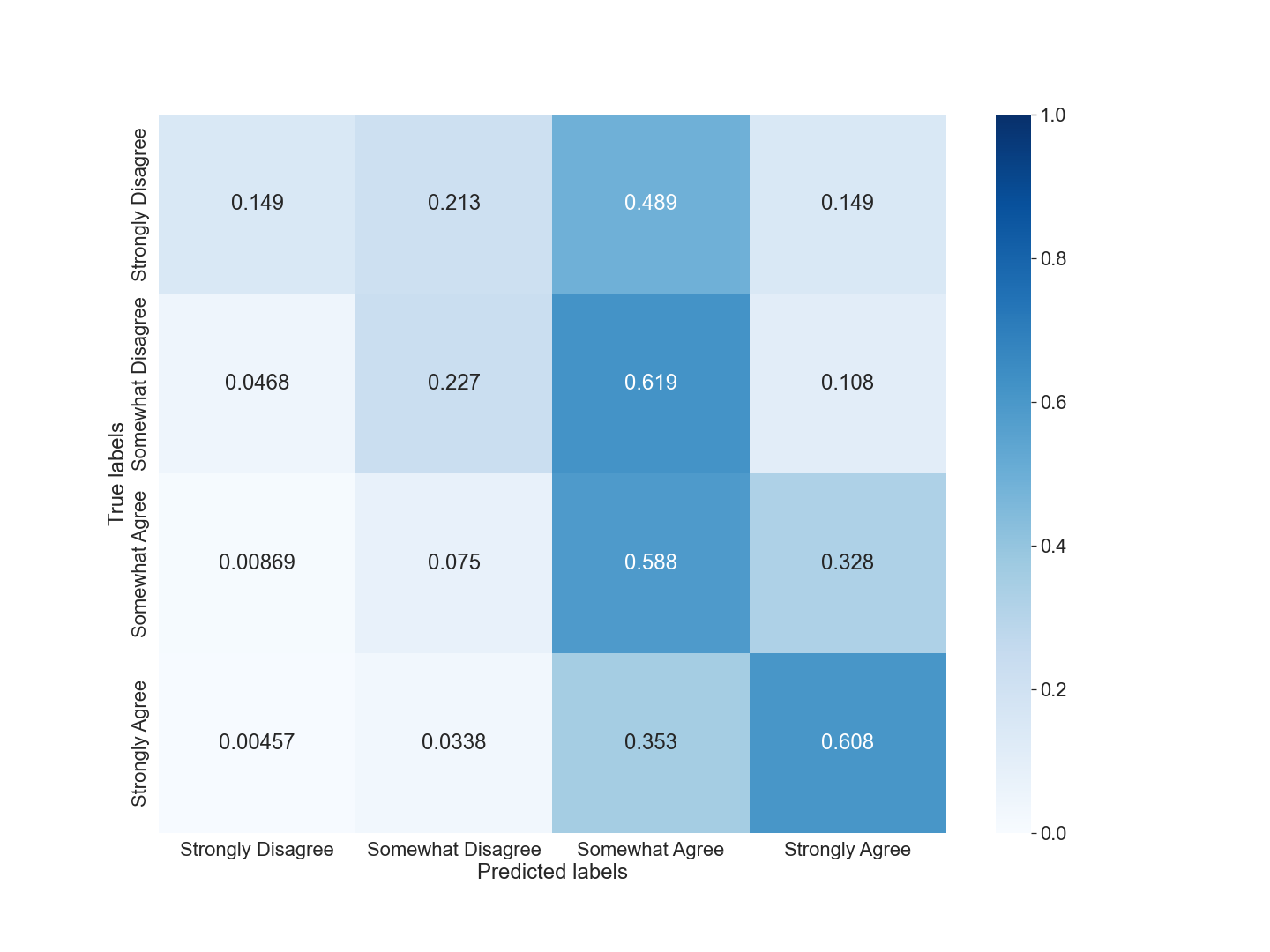


**Figure S1:** Confusion matrix for Model 1 with elements normalized to the true prediction class population sizes. The main diagonal cells indicate predictions that match the true labels. n_1_ = 13661.

**
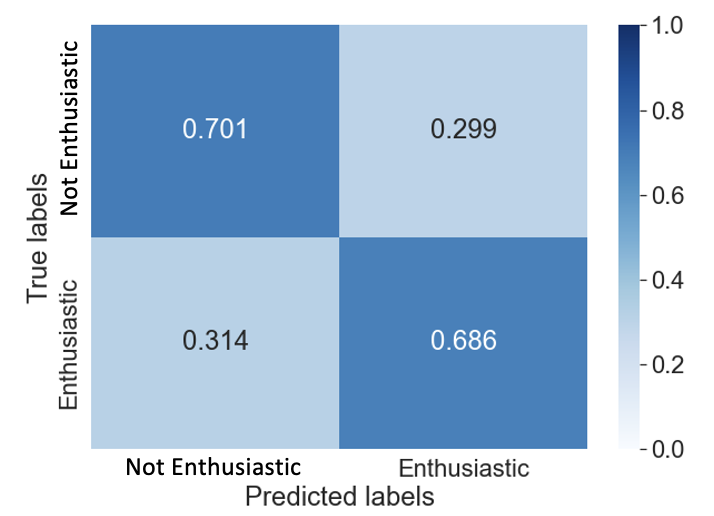
Figure S2:** Confusion matrix for Model 2 with elements normalized to the true prediction class population sizes. The main diagonal cells indicate predictions that match the true labels. n_2_ = 13661.


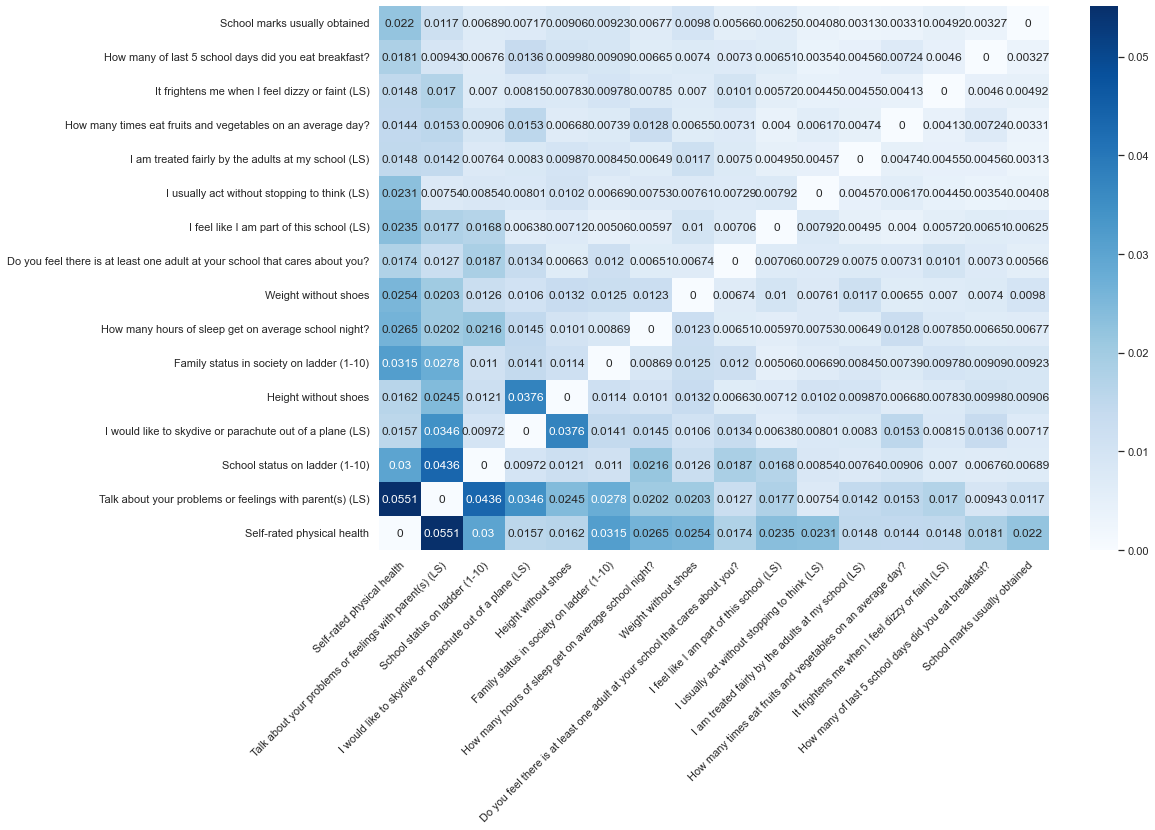


**Figure S3:** SHAP Interaction heatmap for the top 15 Variables in Model 1 (Multiclass Classification), Class 0 (Strongly Disagree). n_1_ = 13661. (LS = Likert Scale).


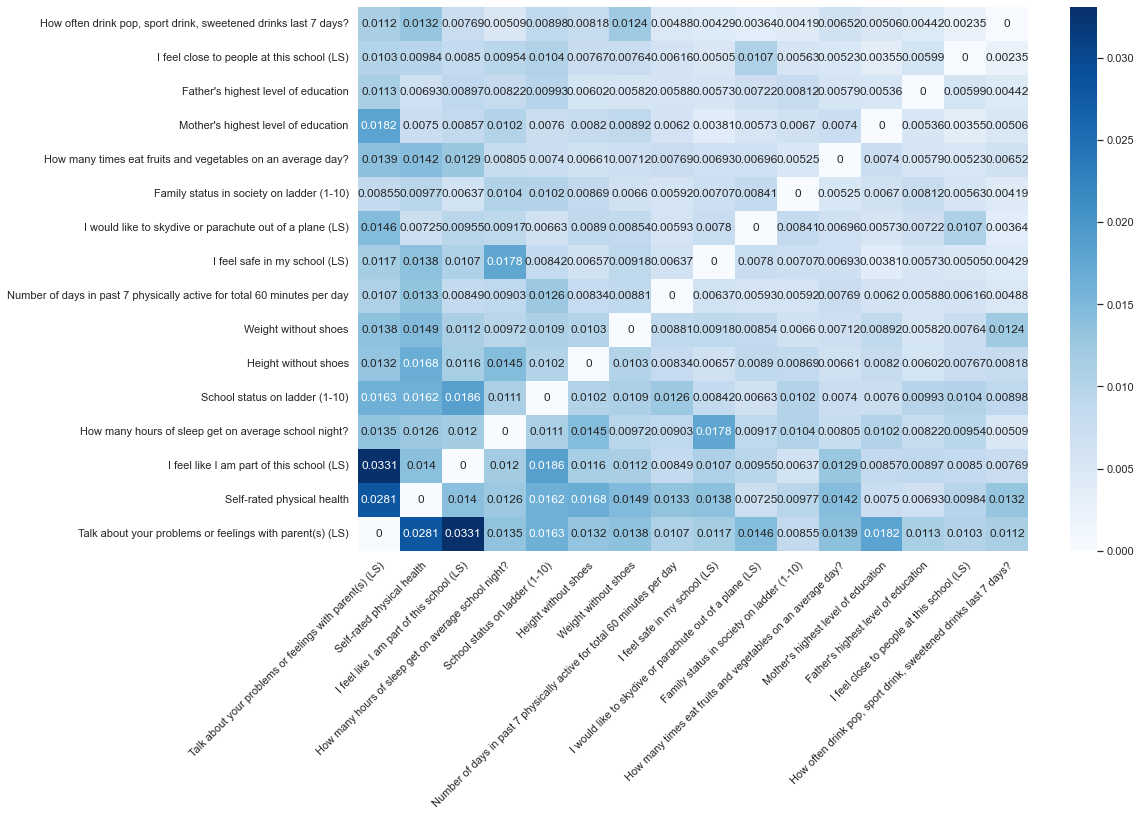


**Figure S4:** SHAP Interaction heatmap for the top 15 Variables in Model 1 (Multiclass Classification), Class 1 (Somewhat Disagree). n_1_ = 13661. (LS = Likert Scale).

**
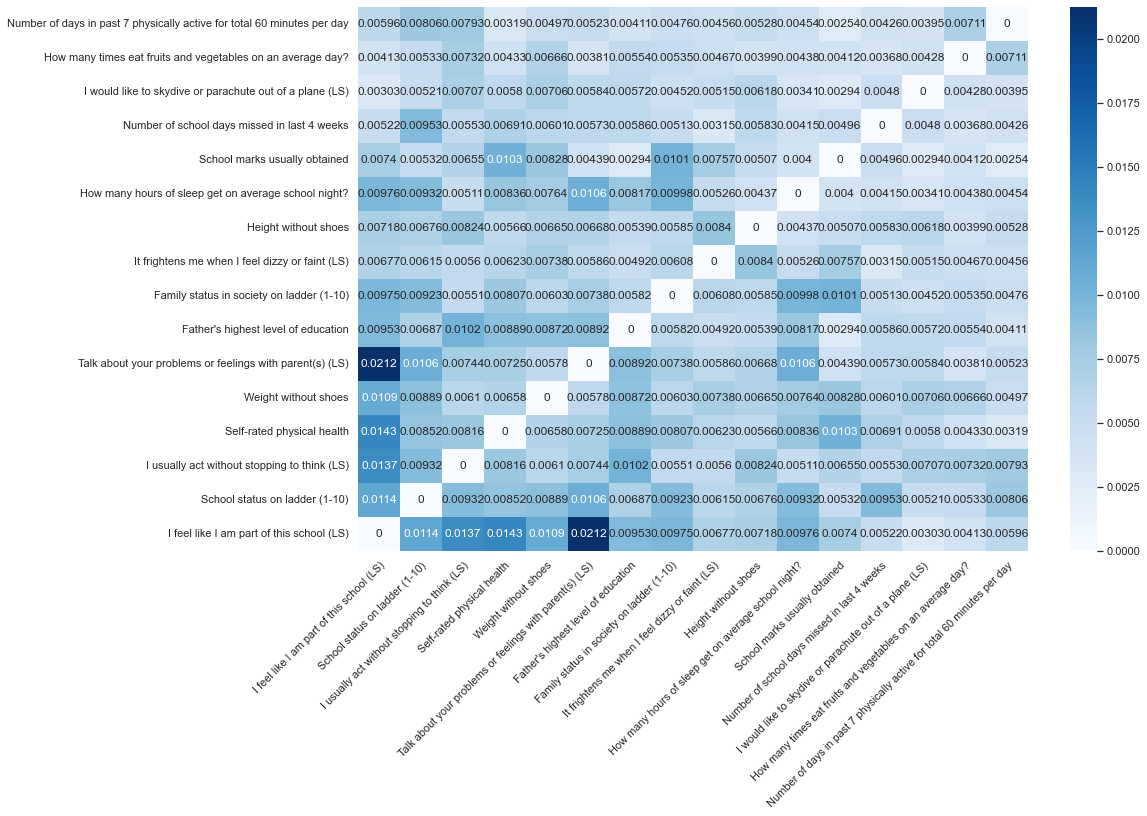
Figure S5:** SHAP Interaction heatmap for the top 15 Variables in Model 1 (Multiclass Classification), Class 2 (Somewhat Agree). n_1_ = 13661. (LS = Likert Scale).


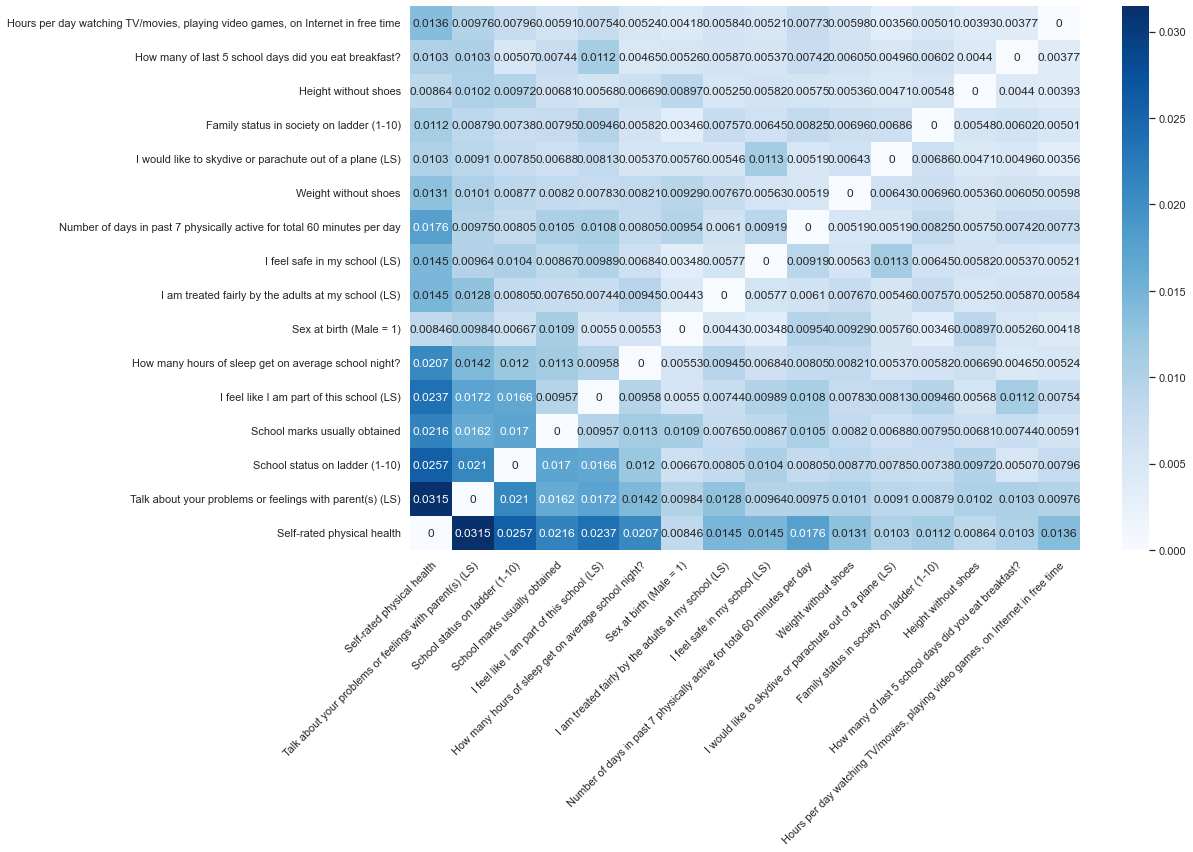


**Figure S6:** SHAP Interaction heatmap for the top 15 Variables in Model 1 (Multiclass Classification), Class 3 (Strongly Agree). n_1_ = 13661. (LS = Likert Scale).

**
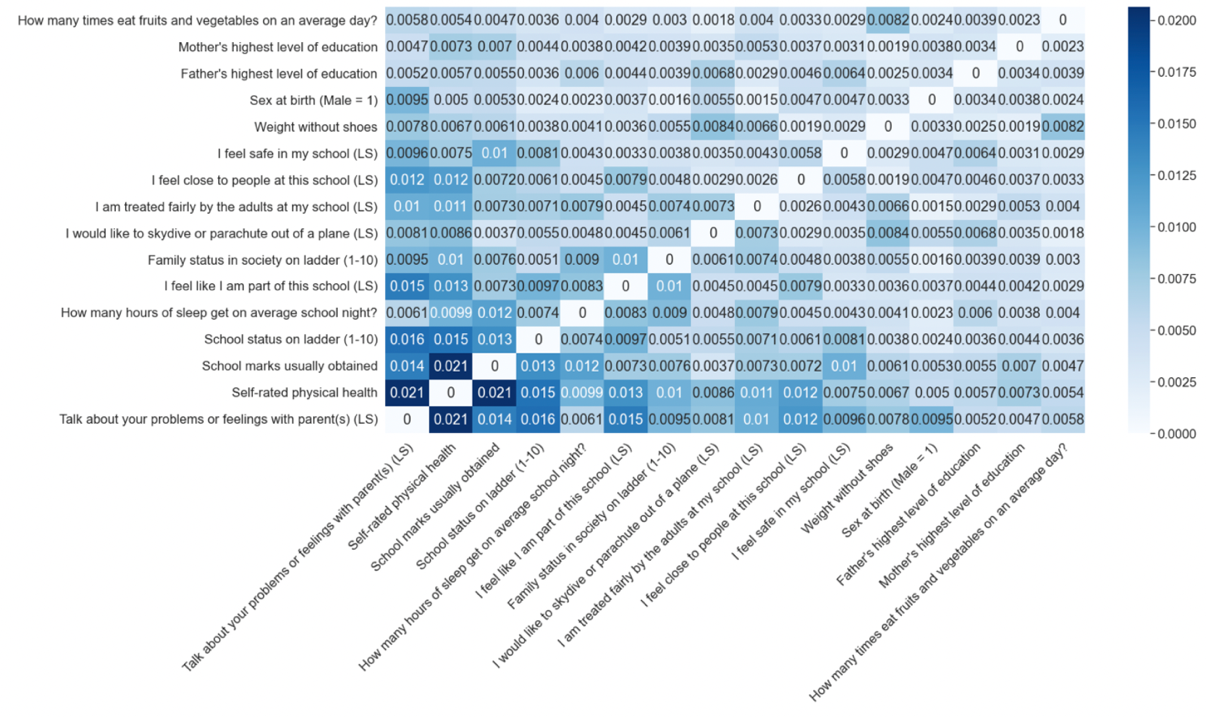
**

**Figure S7:** SHAP Interaction heatmap for the top 15 Variables in Model 2 (*Binary classification (enthusiastic vs. all others)*). n_2_ = 13661. (LS = Likert Scale).

###
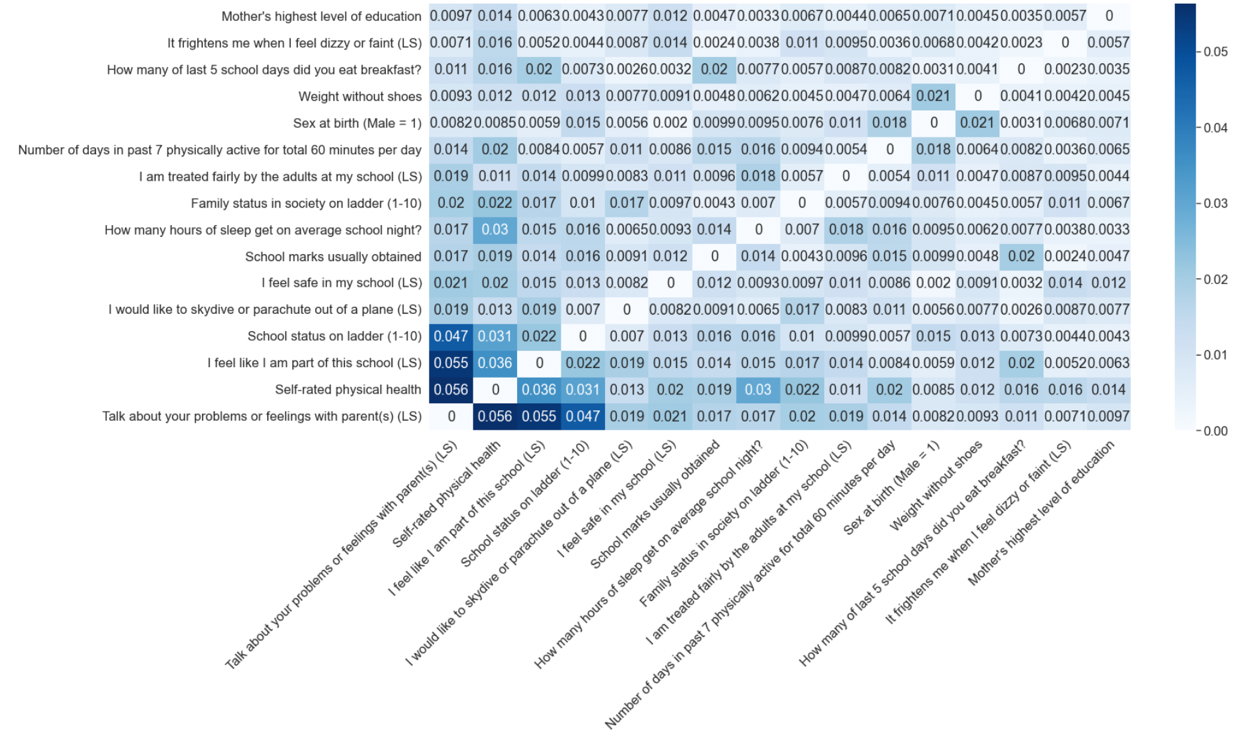


**Figure S8:** SHAP Interaction heatmap for the top 15 Variables in Model 3 (*Binary classification (enthusiastic vs. not enthusiastic)*). n_3_ = 7330. (LS = Likert Scale).

## Supplementary Results

### Section A: Cronbach Alpha

Cronbach’s alpha measure of internal consistency for our set of 50 input features was 0.425. This was calculated in the interest of transparency. However, it’s important to note that a higher Cronbach’s alpha would not reflect a desirable quality of our data set because features were not selected based on their coherence and include variables across several domains violating the unidimensionality assumption of Cronbach’s alpha.

### Section B: Sensitivity Analysis

Sensitivity analysis was completed including only those who initially reported strong agreement or disagreement with “I feel enthusiastic for the future” (removing students who responded either somewhat agree or disagree). Initially, as described in the main text, only the “somewhat agree” responses were removed from analysis as this preserved enough respondents, removed those who could be considered ambivalent, and enabled comparison between those who were very enthusiastic and those who answered negatively. There is a low percentage (470 (7.91%) students versus 5472 (92.1%) in the sensitivity analysis) of respondents in the ‘strongly disagree’ class. This would have led to an increased oversampling of ‘strongly disagree’ group when balancing the classes prior to classification, leading to increased overfitting and accuracy of the model. With both groups excluded, the accuracy improved (from 81% to 92%) though the top five important variables as measured by SHAP analysis did not change.

**Table S5:** Performance metrics calculated for sensitivity analysis on withheld test data.

|  | **n Train (80% of data)** | **n Test (20% of data)** | **AUROC** | **Accuracy** [95% CI] | **Precision** [95% CI] | **Recall** [95% CI] |
| --- | --- | --- | --- | --- | --- | --- |
| *Sensitivity Analysis* | 4753 | 1189 | 0.89 | 0.92 [0.90, 0.94] | 0.91 [0.89, 0.93] | 0. 92 [0.90, 0.94] |


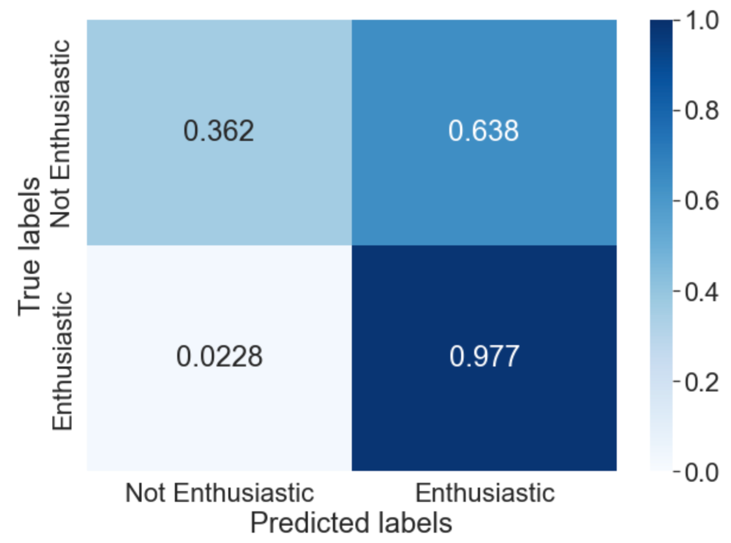


**Figure S9:** Confusion matrix for Sensitivity Analysis with elements normalized to the true prediction class population sizes. The main diagonal cells indicate predictions that match the true labels. n_SA_ = 5942.

**Table S6:** Top five variables identified by magnitude of importance (rank).

| **Rank** | *Sensitivity Analysis* | |
| --- | --- | --- |
|  | **Input Feature** | **Mean Absolute SHAP Value** |
| **1** | Self-rated physical health | 0.611 |
| **2** | Feeling comfortable sharing one’s thoughts or feelings with their parents | 0.506 |
| **3** | Feeling a sense of belonging in the school community | 0.303 |
| **4** | Perceived school status on a scale of 1-10 | 0.286 |
| **5** | School marks usually obtained | 0.240 |
